# A Bibliometric Analysis of Clinical Trials on Salivary Biomarkers for Mental Health (2003-2024)

**DOI:** 10.7759/cureus.64635

**Published:** 2024-07-16

**Authors:** Namrata Dagli, Mainul Haque, Santosh Kumar

**Affiliations:** 1 Department of Research, Karnavati Scientific Research Center, Karnavati School of Dentistry, Karnavati University, Gandhinagar, IND; 2 Department of Research, Karnavati Scientific Research Center, School of Dentistry, Karnavati University, Gandhinagar, IND; 3 Department of Pharmacology and Therapeutics, National Defence University of Malaysia, Kuala Lumpur, MYS; 4 Department of Periodontology and Implantology, Karnavati School of Dentistry, Karnavati University, Gandhinagar, IND

**Keywords:** non-invasive biological fluid, biblioshiny, vosviewer, stress, psychological status, mental health, salivary biomarker, pubmed, bibliometric study, scientometric study

## Abstract

Mental health conditions, such as depression, anxiety, and stress-related disorders, are often difficult to diagnose and monitor using traditional methods. Salivary biomarkers offer a promising alternative due to their non-invasive nature, ease of collection, and the potential to reflect real-time physiological changes associated with mental health. This bibliometric analysis examines 95 clinical trials on stress biomarkers for mental health, published between 2003 and 2024. The field is characterized by extensive collaboration and global participation, involving 593 authors and publications across 73 journals. Despite a consistent annual publication rate, notable increases in 2011, 2014, and 2018 indicate growing research interest. The United States leads in research output, followed by Australia, Germany, and Japan, with Psychoneuroendocrinology being the most prominent journal. Co-occurrence analysis identified nine research clusters, suggesting diverse directions such as the impact of stress-related hormones, circadian rhythms, mindfulness, various therapies, aging, psychological adaptation mechanisms, exercise therapy, anxiety disorders, and the autonomic nervous system on salivary biomarkers. Key terms such as "biomarkers/metabolism," AND "hydrocortisone/metabolism," AND "saliva/metabolism" were central, with significant activity from 2012 to 2018. This analysis highlights a growing focus on the metabolic processes and therapeutic applications of salivary biomarkers in mental health. This bibliometric analysis calls attention to the promising potential of salivary biomarkers to revolutionize mental health diagnostics and treatment through non-invasive methods, fostering interdisciplinary research, technological advancements, and global health improvements.

## Introduction and background

The exploration of salivary biomarkers has emerged as a promising frontier in the quest to enhance the diagnosis and monitoring of mental health disorders. Saliva, a readily accessible and non-invasive biological fluid, offers a unique window into the body's physiological state, reflecting various physiological and biochemical systemic changes associated with mental health conditions [1,2]. The ability to detect and measure biomarkers in saliva presents an attractive alternative to traditional diagnostic methods, which often involve more invasive procedures such as blood draws or cerebrospinal fluid collection [3]. In recent years, the field has witnessed a growing interest in identifying and validating salivary biomarkers for a range of mental health disorders, including stress, depression, anxiety, schizophrenia, and bipolar disorder [4-11]. These biomarkers can include hormones, enzymes, antibodies, and other molecular entities that correlate with pathological changes in the brain and body associated with these conditions [12,13].

Key salivary biomarkers studied in the context of mental health include cortisol, alpha-amylase, and various inflammatory markers [13]. Cortisol, a stress hormone, is often measured to assess the hypothalamic-pituitary-adrenal (HPA) axis function, which is commonly dysregulated in disorders such as depression and anxiety. Elevated or blunted cortisol responses can indicate chronic stress and maladaptive stress responses, which are critical in understanding and managing these conditions. Alpha-amylase, an enzyme linked to the sympathetic nervous system, is another marker for stress and arousal. Its levels in saliva can indicate acute stress responses and have been studied concerning anxiety and other stress-related disorders. Inflammatory markers in saliva, such as cytokines, also provide insights into the inflammatory processes that may underpin various mental health disorders. Chronic inflammation has been implicated in the pathophysiology of depression, schizophrenia, and bipolar disorder, making these markers valuable for both research and potential clinical applications [13-16].

The use of salivary biomarkers in mental health research and clinical practice offers several advantages, including ease of collection, the possibility for repeated measures, and minimal discomfort for patients. These characteristics make saliva an attractive medium for longitudinal studies and monitoring treatment responses. The study of salivary biomarkers holds promise for advancing our understanding of mental health disorders, improving diagnostic accuracy, and enabling personalized treatment approaches. As research in this field progresses, it may lead to the development of novel, noninvasive diagnostic tools that can significantly enhance mental health care [17,18].

A bibliometric analysis of clinical trials focusing on salivary biomarkers for mental health is essential to map the research landscape in this area. This analysis provides valuable insights into the evolution of scientific inquiry, the intensity of research efforts, collaboration networks, and emerging trends. By examining publication patterns and the geographical distribution of research activities, we can identify critical contributors, collaborators, and potential gaps in the current knowledge base.

This bibliometric study aims to systematically evaluate clinical trial literature on salivary biomarkers for mental health. Through this analysis, we strive to illuminate the trajectory of research developments, highlight significant findings, and underscore the potential of salivary diagnostics in revolutionizing mental health care. Ultimately, this work seeks to inform future research directions and support the integration of salivary biomarkers into clinical practice, enhancing early detection, personalized treatment, and monitoring of mental health disorders.

## Review

Material and methods

Data Source and Search Strategy

A comprehensive bibliometric analysis was conducted on clinical trials related to salivary biomarkers for mental health. The primary database utilized for this study was PubMed, a widely recognized and authoritative source for biomedical literature. The search was conducted on June 15, 2024, using specific keywords and Medical Subject Headings (MeSH) terms related to salivary biomarkers and mental health disorders. The following search string was used to capture a broad range of relevant studies - (salivary biomarkers) AND ("mental health" OR "mental disorder" OR "psychiatric disorder" OR "psychological stress" OR "psychological depression" OR "mood disorder" OR "anxiety" OR "Schizophrenia" OR "bipolar disorder") AND (Human) NOT (animal).

Inclusion and Exclusion Criteria

To ensure the relevance and quality of the studies included in this analysis, we applied the following inclusion and exclusion criteria (Table 1). 

**Table 1:** Depicting inclusion and exclusion criteria.

Inclusion Criteria	Exclusion Criteria
Clinical trials	Animal studies
Studies published in English	Non-English publications
Studies involving human subjects	Studies other than clinical trials (e.g., reviews, observational studies, meta-analyses)

Study Selection Process

The study selection process adhered to the Preferred Reporting Items for Systematic Reviews and Meta-Analyses (PRISMA) guidelines [19]. The data were exported from PubMed to a text file. Two independent reviewers screened the titles and abstracts of the retrieved articles to confirm the inclusion of only relevant studies. Any disagreements with the reviewer were resolved through discussion or consultation with a third reviewer.

Data Extraction and Analysis

Bibliometric data, including publication year, journal, authors, country of origin, and sources, were extracted from the included studies. The extracted data were analyzed using the following software tools.

VOSviewer visualizes and analyzes bibliometric networks, including coauthorship and keyword co-occurrence [20]. Biblioshiny (R Development Core Team, Vienna, Austria), an R-based software tool, provides comprehensive capabilities for bibliometric analysis and visualization [21]. Microsoft Excel manages data, cleans it, and conducts basic descriptive statistics. BioRender creates high-quality scientific figures and diagrams [22].

Results

Search Results

**Figure 1:**
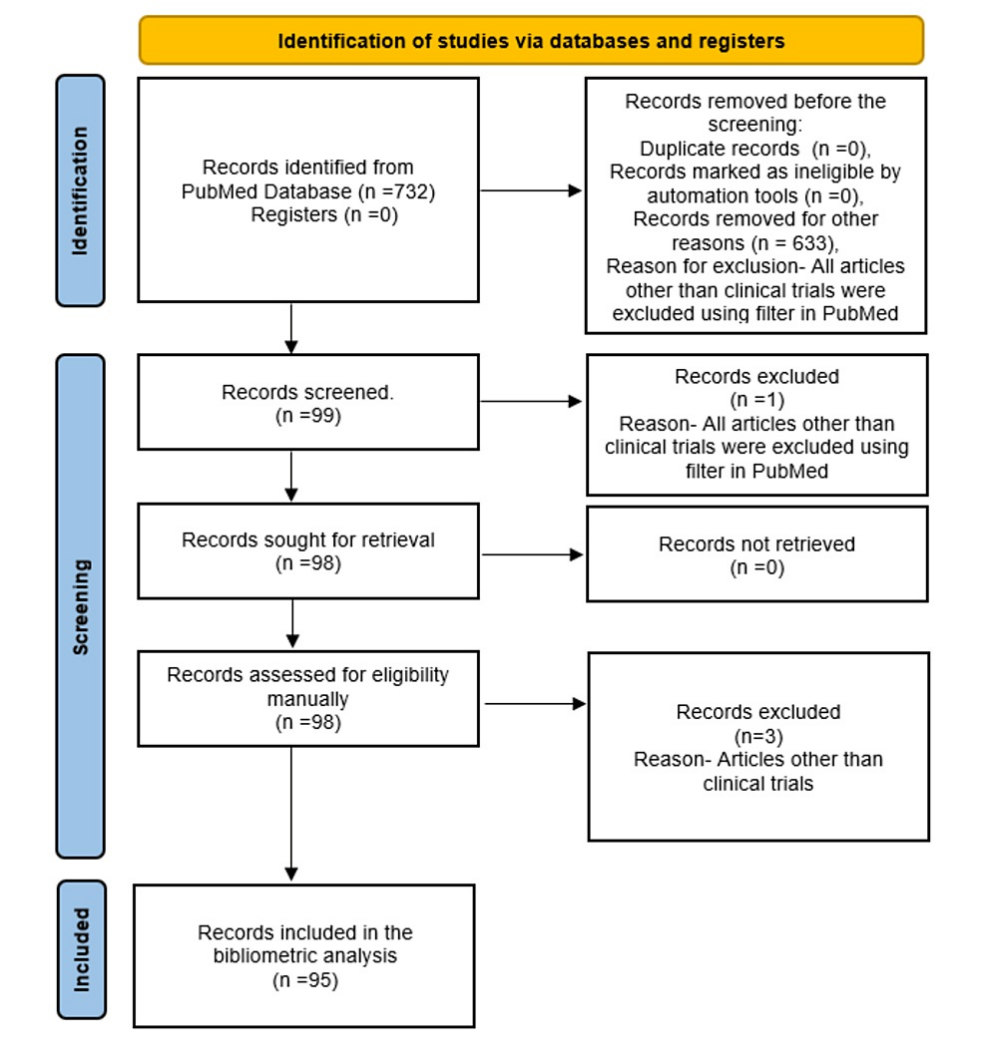
PRISMA flow chart of the study selection process for the bibliometric analysis on salivary biomarkers of mental health. PRISMA: Preferred Reporting Items for Systematic Reviews and Meta-Analyses Image Credit: Namrata Dagli.

A PubMed search yielded 732 articles, of which 99 were identified as clinical trials. One non-English article was excluded, leaving 98 articles for manual screening to verify the results. After excluding three study protocols, 95 clinical trials published in English were selected for this bibliometric analysis (Figure 1).

Main Information

The Biblioshiny app identified that the dataset on clinical trials investigating salivary biomarkers in the context of mental health spans from 2003 to 2024. It comprises 95 documents sourced from 73 different journals, books, and other publications. The documents have an average age of 8.65 years, suggesting a relatively recent interest in this research area. Regarding the content of the documents, there are 621 keywords, reflecting various terms used to describe the research focus. The authorship data show involvement from 593 different authors, with only one document being single-authored. This signifies a highly collaborative field, supported by an average of 6.48 co-authors per document.

Furthermore, 14.74% of the documents involve international coauthorship, highlighting a significant level of global collaboration in this research area. Overall, the data suggest that very few clinical trials have been done on salivary biomarkers for mental health. The trials are characterized by collaborative, international efforts, exploring various topics over the past two decades.

Annual Scientific Publication

**Figure 2:**
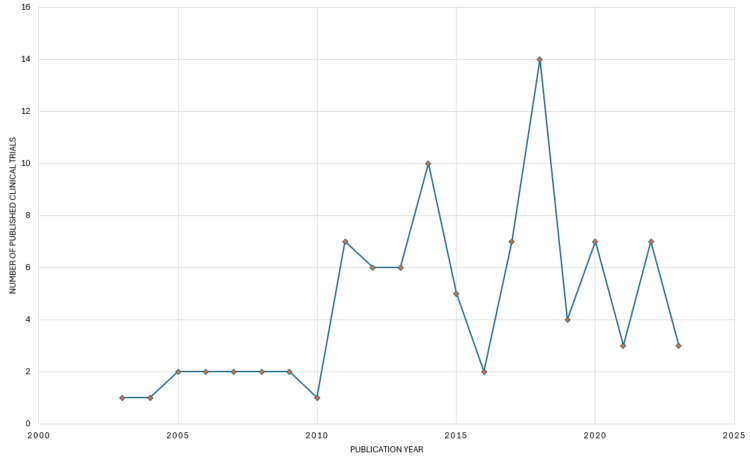
Annual scientific publications on salivary biomarkers for mental health in PubMed. Image Credit: Namrata Dagli.

The number of published clinical trials on salivary biomarkers for mental health has shown significant variation over the years (Figure 2). From 2003 to 2010, the number of articles published annually remained relatively low and stable, with a maximum of two articles per year. However, there was a notable increase in 2011, with the number of published articles jumping to seven and maintaining a higher activity level through the subsequent years. Specifically, 2012 saw a slight decrease to six articles, but the number of publications remained relatively high, with another peak in 2014 at 10 articles. After a dip in 2015 with five articles, the publication rate dropped significantly in 2016 to two articles but surged again in 2017 with seven articles. The most substantial increase occurred in 2018, with 14 articles published indicating a growing interest and research activity in this field. This was followed by a decline in 2019 with four articles, a rise again in 2020 with seven articles, a slight drop in 2021 to three articles, a return to seven articles in 2022, and finally three articles published in 2023. Overall, the data reveal an upward trend in research publications from 2011 onwards, with notable peaks and troughs reflecting fluctuating interest and advancements in studying salivary biomarkers of mental health.

Coauthorship Analysis of Authors

**Figure 3:**
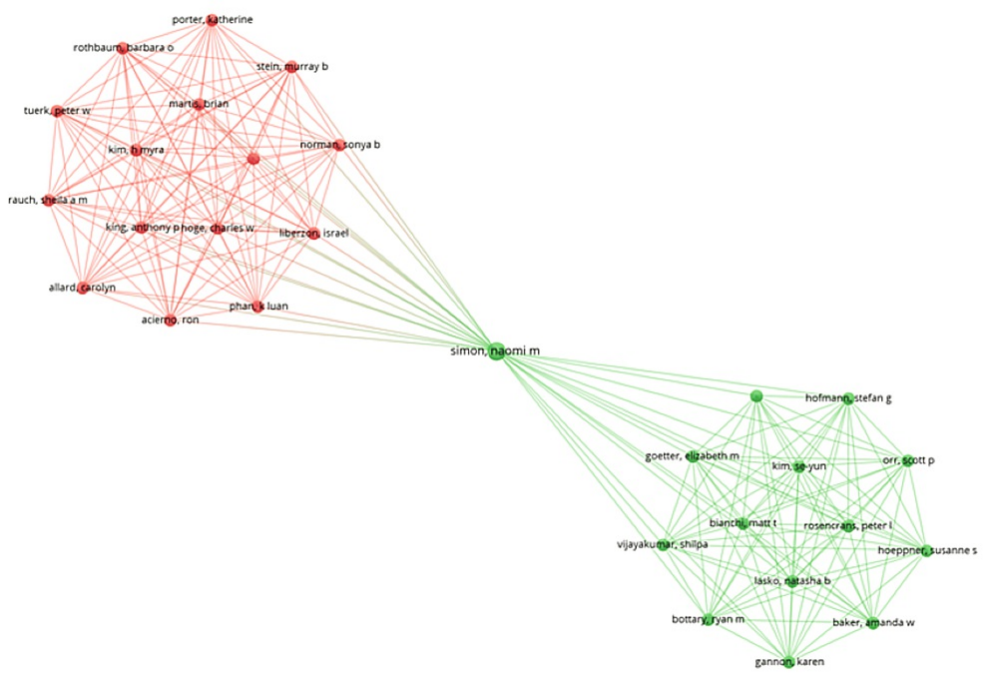
Coauthorship analysis of authors of clinical trials on salivary biomarkers of mental health. Image Credit: Namrata Dagli

The network visualization was created using VOSviewer, identifying 595 authors who have published at least one clinical trial on salivary biomarkers of mental health. The strength of the coauthorship links was calculated for each of these 595 authors. Since not all authors were connected, the visualization includes only the largest group of 29 connected authors, spread across two clusters with 211 links (Figure 3). Naomi M. Simon was identified as having the highest total link strength (TLS) of coauthorship with a value of 28, followed by George P. Chrousos and Con Stough, each with a TLS value of 20.

Most Relevant Countries

**Figure 4:**
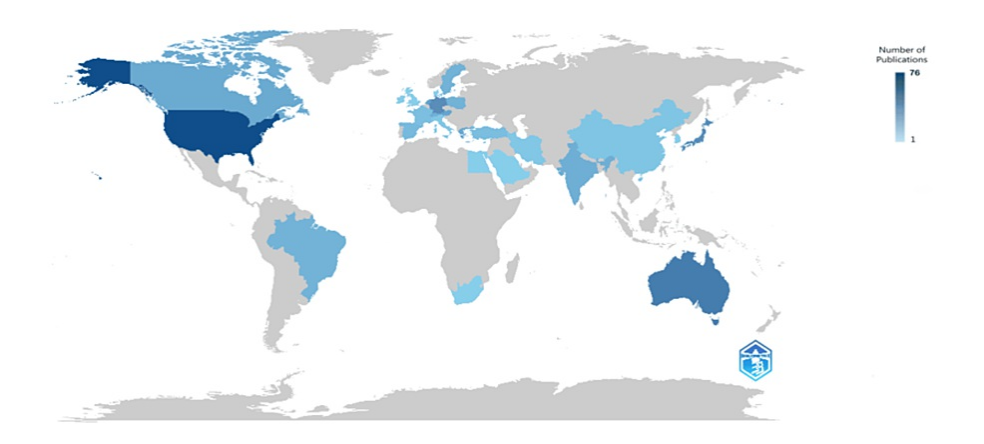
Scientific production of countries. Image Credit: Namrata Dagli

The number of published clinical trials on salivary biomarkers of mental health varies significantly across different countries. The United States leads with a substantial margin, boasting 76 clinical trials. Australia follows as the second-highest, with 47 trials, indicating a strong research interest in this area within the country. Germany and Japan have comparable numbers, with 37 and 36 trials, respectively, showing a similar level of engagement in this research field. The Netherlands and Austria contribute moderately, with 24 and 23 trials, respectively. Canada and Greece each have 21 published trials, reflecting a balanced focus on salivary biomarkers of mental health. India and Switzerland have 18 trials, suggesting a developing but notable interest in this topic. These figures highlight the global distribution and varying levels of research activity in studying salivary biomarkers and their relation to mental health across these countries (Figure 4).

Most Relevant Journals

**Figure 5:**
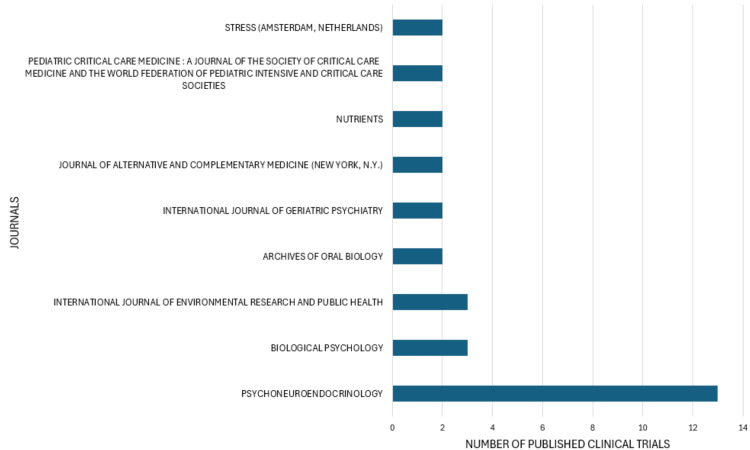
Most relevant journals based on the number of clinical trials published on salivary biomarkers of mental health. Image Credit: Namrata Dagli

A significant disparity becomes apparent when examining the number of published clinical trials on salivary biomarkers of mental health across various sources. The journal Psychoneuroendocrinology leads with a notable 13 articles, reflecting its specialized focus on the intersection of psychological processes and endocrine function, making it a prominent source in this niche research area. In contrast, Biological Psychology and the International Journal of Environmental Research and Public Health each contribute three articles indicating moderate interest and research activity. Several other journals, including the Archives of Oral Biology, International Journal of Geriatric Psychiatry, Journal of Alternative and Complementary Medicine, Nutrients, Pediatric Critical Care Medicine, and Stress, each feature two articles, highlighting a broader but less concentrated interest in the topic across these fields. This distribution underscores a strong emphasis in specific specialized journals while indicating a more diffuse and less frequent exploration in others (Figure 5).

Co-occurrence Analysis of Keywords

**Figure 6:**
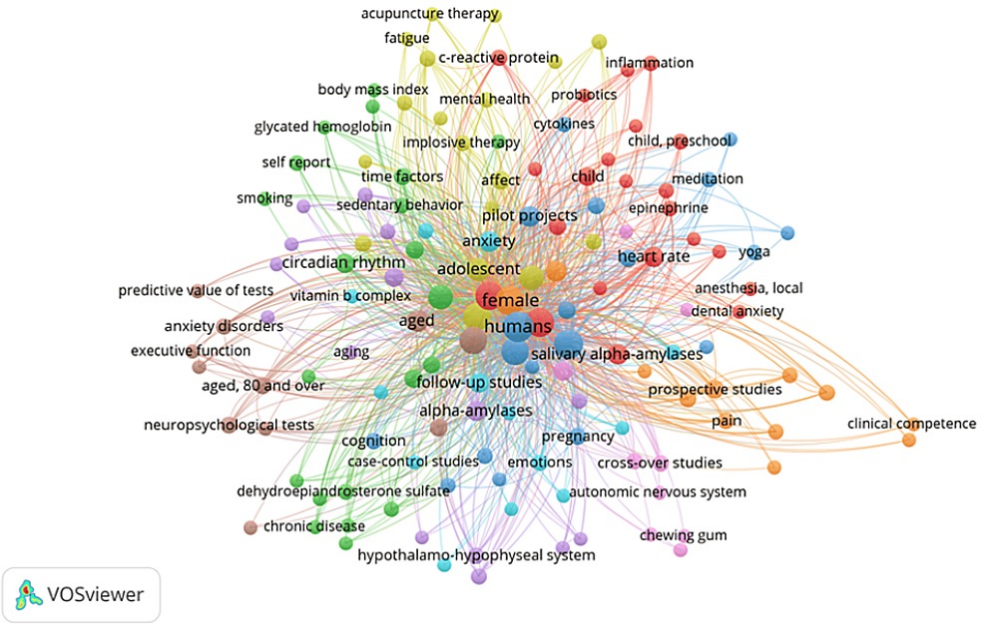
Co-occurrence analysis of keywords identified in clinical trials published in PubMed on salivary biomarkers on oral health. Image Credit: Namrata Dagli

Using VOSviewer, 446 MeSH keywords were identified, 140 of them appearing twice. The network visualization (Figure 6) includes these 140 keywords, distributed across nine clusters, with 2,317 links and a total link strength of 6,583. The keywords in each cluster are listed in Table 2.

**Table 2:** Keywords in each cluster identified in the co-occurrence analysis of keywords.

Clusters	Keywords
Cluster 1 (22 items)	Anesthesia local, biomarkers, blood pressure, c-reactive protein, child, child preschool, dental anxiety, epinephrine, healthy volunteers, heart rate, inflammation, infusions intravenous, male, norepinephrine, poverty, probiotics, psychometrics, salivary alpha-amylases, sensitivity and specificity, single-blind method, statistics nonparametric, students
Cluster 2 (21 items)	Analysis of variance, arteriosclerosis, body mass index, chronic disease, circadian rhythm, dehydroepiandrosterone sulfate, depression, glycated hemoglobin, health behavior, middle-aged, obesity, reference values, regression analysis, risk factors, sedentary behavior, self-report, severity of illness index, sex factors, smoking, testosterone, time factors.
Cluster 3 (19 items)	Adult, breast neoplasms, cancer survivors, cocaine-related disorders, cognition, cytokines, enzyme-linked immunosorbent assay, feasibility studies, humans, interleukin- 10, interleukin-6, meditation, mindfulness, pilot projects, pregnancy, prenatal care, quality of life, stress psychological, yoga
Cluster 4 (18 items)	Acupuncture therapy, adolescent, affect, cognitive behavioral therapy, fatigue, health promotion, hydrocortisone, imagery psychotherapy, implosive therapy, mental health, outcome assessment health care, perception, phobia social, psychotherapy, psychotherapy group, sleep, wakefulness, young adult
Cluster 5 (15 items)	Adrenergic beta-antagonists, age factors, aging, alpha-amylases, Alzheimer's disease, antioxidants, cardiovascular diseases, dietary supplements, double-blind method, hypothalami-hypophyseal system, pituitary-adrenal system, plant extracts, propranolol, sex characteristics, sympathetic nervous system
Cluster 6 (13 items)	Adaptation psychological, anxiety, case-control studies, emotions, follow-up studies, neoplasms, oligopeptides, patient education as a topic, prognosis, psychiatric status rating scale, reproducibility of results, specimen handling, vitamin B complex
Cluster 7 (12 items)	Clinical competence, exercise therapy, female, infant newborn, infant premature, pain, pain management, pain measurement, prospective studies, simulation training, stress physiological, treatment outcome
Cluster 8 (11 items)	Aged, aged 80 and over, anxiety disorders, chromogranin a, dementia, executive function, memory, neuropsychological tests, oils volatile, the predictive value of tests, saliva
Cluster 9 (9 items)	The autonomic nervous system, chewing gum, cross-over studies, immunoglobulin a, magnetic resonance imaging, mastication, odorants, rest, surveys, and questionnaires

The co-occurrence analysis of keywords suggests various research directions regarding salivary markers for mental health. Cluster 1 could focus on exploring how salivary alpha-amylases and other biomarkers respond to stressors such as dental anxiety, particularly in children and adults. The relationship between inflammation markers such as C-reactive protein and salivary biomarkers in stress and anxiety contexts can also be investigated. There is also a potential to evaluate the sensitivity and specificity of salivary markers using psychometric tools and examine the impact of stress-related hormones like epinephrine and norepinephrine on these markers.

Cluster 2 suggests investigating the impact of circadian rhythms on salivary markers related to stress and mental health. Research could also explore the relationship between chronic diseases, such as obesity and arteriosclerosis, and salivary biomarkers in mental health. The association between depression and specific salivary markers, such as dehydroepiandrosterone (DHEA) sulfate, is another area of interest, alongside examining how lifestyle factors such as smoking and sedentary behavior influence these biomarkers.

In Cluster 3, research could focus on how stress and psychological factors affect salivary biomarkers in cancer survivors and the role of cytokines such as interleukin-6 and interleukin-10 in mental health. The impact of mindfulness and meditation practices on salivary markers and the correlation between quality of life measures and these biomarkers are also significant areas to explore.

Cluster 4 points to investigating how various therapies, including cognitive behavioral therapy and acupuncture, affect salivary markers in adolescents and adults. The relationship between sleep patterns, wakefulness, salivary biomarkers, and the impact of hydrocortisone levels on these markers in the context of mental health are essential research directions. Another critical area of interest is assessing the effectiveness of different mental health interventions on salivary biomarkers.

Cluster 5 suggests that research could involve investigating the effects of aging on salivary biomarkers, particularly concerning Alzheimer's disease and cardiovascular health. The influence of medications, such as adrenergic beta-antagonists, on these biomarkers and the impact of dietary supplements and antioxidants are essential to explore. Additionally, examining the role of the hypothalamo-hypophyseal and pituitary-adrenal systems in regulating salivary biomarkers is crucial.

Cluster 6 highlights the investigation of how psychological adaptation mechanisms affect salivary biomarkers in anxiety and stress contexts. The correlation between emotional states and these biomarkers, the role of patient education in managing stress, and ensuring the reliability and reproducibility of salivary biomarker assessments are significant research areas.

Cluster 7 suggests examining the effects of exercise therapy on salivary biomarkers related to stress and mental health, as well as the relationship between pain management strategies and these biomarkers. Research could also focus on how physiological stress in newborns and premature infants is reflected in salivary biomarkers, and prospective studies could be conducted to assess treatment outcomes on these biomarkers.

Cluster 8 indicates investigating how anxiety disorders in older adults impact salivary biomarkers and studying the relationship between dementia, memory, and these biomarkers. Exploring the role of chromogranin A as a salivary biomarker for mental health in elderly populations and assessing the predictive value of salivary biomarkers in diagnosing and monitoring mental health conditions are also important research areas.

Finally, Cluster 9 suggests investigating the impact of the autonomic nervous system on salivary biomarkers and studying how chewing gum and mastication influence these markers related to stress and mental health. Research could explore the role of immunoglobulin A in saliva as a marker for mental health and assess how rest and relaxation affect salivary biomarkers. Utilizing surveys and questionnaires to correlate subjective mental health measures with salivary biomarkers is another significant area of focus.

Overall, these clusters provide a comprehensive roadmap for future research on the role of salivary markers in mental health, highlighting the importance of various physiological, psychological, and lifestyle factors.

Analysis of Topic Trends

In analyzing clinical trials on salivary biomarkers for mental health, several keywords stand out regarding their frequency and temporal trends. The keyword "biomarkers/metabolism" is the most frequently cited, appearing 31 times, with a significant research focus between 2012 and 2018. This is closely followed by "hydrocortisone/metabolism," noted 29 times, showing strong interest from 2013 to 2018. "Saliva/metabolism" and "biomarkers/analysis" also have high frequencies, cited 26 and 19 times, respectively, with notable activity from 2013 to 2018. The general keyword "biomarkers" appears 15 times, with a peak in recent years, from 2016 to 2021, suggesting a growing interest in this area.

**Figure 7:**
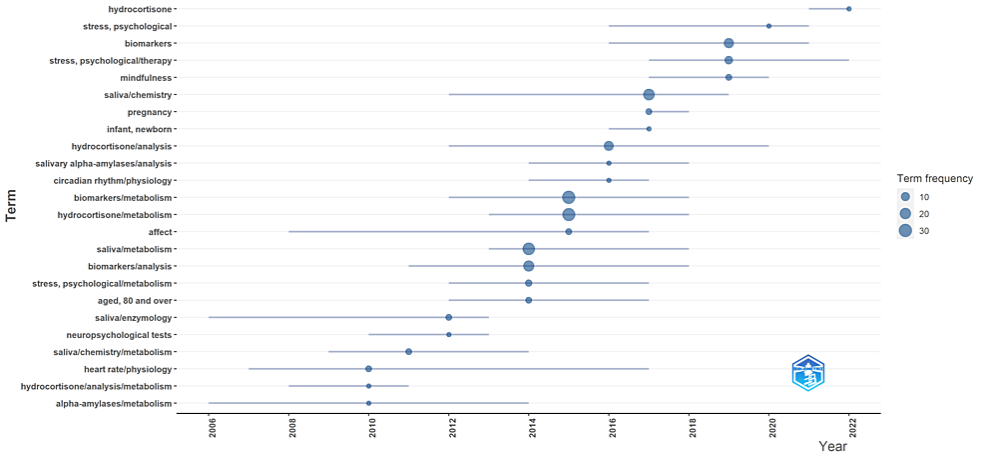
Analysis of topic trends in clinical trials on salivary biomarkers of mental health. Image Credit: Namrata Dagli

Heart rate and alpha-amylases were among the earliest biomarkers studied, with initial peaks in 2007 and 2006, respectively, and continued interest up to 2017 and 2014. Salivary alpha-amylases and hydrocortisone were particularly prominent, reflecting their significant roles in stress and psychological studies. Neuropsychological tests and the physiological study of heart rate also reflect the interdisciplinary approach combining physiological and psychological assessments. Keywords such as "stress, psychological/metabolism" and "stress, psychological/therapy" were consistently present, indicating a focus on understanding and treating psychological stress through biomarkers, with studies increasing from 2012 onward. Research on mindfulness and its impact on stress biomarkers has also emerged prominently in recent years. Overall, the data reveal a comprehensive and evolving landscape of research into salivary biomarkers, with a steady increase in studies focusing on metabolic processes and therapeutic applications in mental health contexts (Figure 7).

Discussion

The bibliometric analysis of clinical trials on stress biomarkers for mental health reveals critical findings from a comprehensive PubMed search, which identified 95 relevant clinical trials published in English between 2003 and 2024. Despite a consistent publication rate, the field is characterized by high collaboration and significant global participation, with involvement from 593 authors across 73 journals and publications. Key trends show a varied annual publication rate, with notable increases in 2011, 2014, and 2018, reflecting growing interest in this research area. The United States leads research output, followed by Australia, Germany, and Japan. This can be attributed to factors such as robust funding, advanced research infrastructure, prominent academic institutions, and robust health policies prioritizing mental health. The United States leads with 76 clinical trials, reflecting its significant investment in mental health research, followed by Australia with 47 trials, driven by public health initiatives. Germany and Japan have comparable outputs due to their healthcare systems and technological advancements. Countries such as the Netherlands, Austria, Canada, Greece, India, and Switzerland also contribute meaningfully, showcasing a global effort to improve mental health diagnostics and treatments through the study of salivary biomarkers. This international research collaboration enhances the understanding and application of these biomarkers across diverse populations and healthcare systems. The most prominent journal in this field is Psychoneuroendocrinology, highlighting its specialized focus on the intersection of psychological and endocrine functions.

A detailed co-occurrence analysis of keywords identified nine research clusters, suggesting diverse research directions such as the impact of stress-related hormones, circadian rhythms, mindfulness practices, various therapies, aging, psychological adaptation mechanisms, exercise therapy, anxiety disorders, and the autonomic nervous system on salivary biomarkers. The analysis also revealed critical keywords such as "biomarkers/metabolism," "hydrocortisone/metabolism," and "saliva/metabolism" as central to the research, with significant activity between 2012 and 2018. This evolving research landscape underscores a growing focus on the metabolic processes and therapeutic applications of salivary biomarkers in mental health contexts.

**Figure 8:**
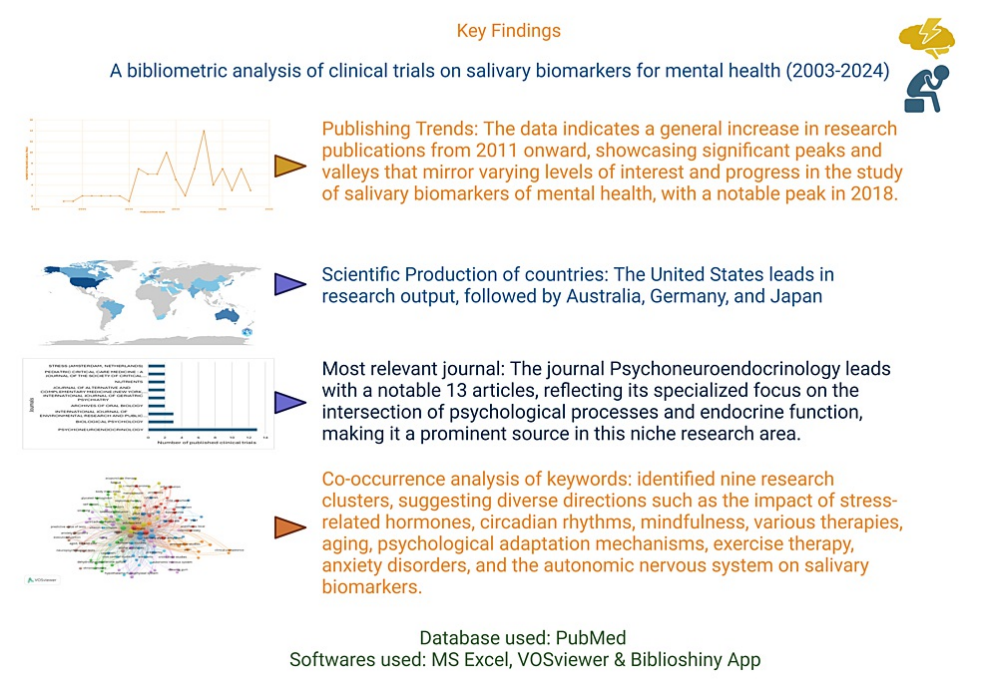
Key findings of the bibliometric analysis of clinical trials on salivary biomarkers for mental health. Notes: This figure was drawn using the Biorender [22] premium version (https://www.biorender.com/), which has the license agreement number ICD270LY1GL. It was accessed on July 3, 2024. Image Credit: Namrata Dagli

One of the strengths of this study lies in its use of the PubMed database, which is widely recognized for its comprehensive coverage of biomedical literature. By leveraging PubMed, the analysis ensures a robust foundation of scholarly articles focused on mental health stress biomarkers, enhancing the bibliometric findings' reliability and breadth. Moreover, the study employs a combination of qualitative and quantitative methodologies, including thematic analysis of keyword co-occurrence. This dual approach quantifies publication trends and author collaborations and identifies and interprets thematic clusters within the literature. By integrating qualitative insights with quantitative metrics, the study provides a nuanced understanding of research themes and directions in stress biomarkers for mental health. This methodological rigor enhances the comprehensiveness and depth of the bibliometric analysis, contributing valuable insights to the scholarly discourse and informing future research endeavors. We have summarized the findings in Figure 8. To our knowledge, no other bibliometric analysis has been done on this topic. However, a bibliometric analysis of salivary biomarkers and a few reviews have been found [23-28].

Limitations of This Study

The bibliometric analysis has its limitations. Firstly, this study exclusively uses data from the PubMed database, which restricts the analysis to publications indexed within this repository. Although PubMed is one of the largest repositories for scientific research papers, excluding other databases may lead to an incomplete representation of the literature on the topic. Additionally, the investigation is primarily quantitative, concentrating on metrics such as publication frequency and authorship patterns without evaluating the quality of individual papers. Consequently, the analysis might overlook significant nuances or variations in research methodologies, findings, and interpretations across different studies. Despite these limitations, the study provides a comprehensive overview of the research landscape of the field, offering valuable guidance for emerging researchers and enhancing understanding of the subject matter.

Future Study Recommendations

While the bibliometric analysis of clinical trials on stress biomarkers for mental health reveals significant insights, several unexplored areas warrant further investigation. Future research should expand beyond cortisol and alpha-amylase to include a broader range of biomarkers, such as DHEA, neuropeptides, and cytokines, and incorporate genetic and epigenetic markers for deeper insights into stress susceptibility. Longitudinal studies are needed to distinguish between acute and chronic stress impacts, as well as those focusing on different life stages. Comparative studies on the efficacy of various interventions, the influence of lifestyle factors, and cross-cultural and population diversity are crucial. Advanced analytical techniques and real-time monitoring technologies, alongside standardized methods and quality assessments of individual studies, will enhance the reliability and comprehensiveness of findings, leading to more effective and personalized mental healthcare solutions.

## Conclusions

The bibliometric analysis of 95 clinical trials on stress biomarkers for mental health from 2003 to 2024 highlights a field marked by high collaboration and significant global participation, with 593 authors and publications across 73 journals. Despite a consistent publication rate, notable increases in 2011, 2014, and 2018 indicate growing interest. The United States leads in research output, followed by Australia, Germany, and Japan, with Psychoneuroendocrinology emerging as the most prominent journal. Co-occurrence analysis of keywords revealed nine research clusters, suggesting diverse directions such as the impact of stress-related hormones, circadian rhythms, mindfulness, various therapies, aging, psychological adaptation mechanisms, exercise therapy, anxiety disorders, and the autonomic nervous system on salivary biomarkers. Key terms such as "biomarkers/metabolism," "hydrocortisone/metabolism," and "saliva/metabolism" were central, with significant research activity from 2012 to 2018, underscoring a growing focus on the metabolic processes and therapeutic applications of salivary biomarkers in mental health.
